# Lyophilization process optimization and molecular dynamics simulation of mRNA-LNPs for SARS-CoV-2 vaccine

**DOI:** 10.1038/s41541-023-00732-9

**Published:** 2023-10-09

**Authors:** Mingyuan Li, Lin Jia, Yanbo Xie, Wenlin Ma, Zhihong Yan, Fufeng Liu, Jie Deng, Ali Zhu, Xue Siwei, Wen Su, Xiaofeng Liu, Shiqin Li, Haomeng Wang, Peng Yu, Tao Zhu

**Affiliations:** 1 China International Science and Technology Cooperation Base of Food Nutrition/Safety and Medicinal Chemistry, Tianjin International Cooperation Research Centre of Food Nutrition/Safety and Medicinal Chemistry, College of Biotechnology, Tianjin University of Science & Technology, Tianjin, 300457 China; 2 CanSino Biologics Inc., Tianjin, 300301 China; 3 CanSino (Shanghai) Biotechnologies Co., Ltd, Shanghai, 201208 China; 4 CanSino (Shanghai) Biological Research Co., Ltd, Shanghai, 201208 China

**Keywords:** Infectious diseases, Viral infection

## Abstract

Some studies have shown that lyophilization significantly improves the stability of mRNA-LNPs and enables long-term storage at 2–8 °C. However, there is little research on the lyophilization process of mRNA-lipid nanoparticles (LNPs). Most previous studies have used empirical lyophilization with only a single lyoprotectant, resulting in low lyophilization efficiency, often requiring 40–100 h. In the present study, an efficient lyophilization method suitable for mRNA-LNPs was designed and optimized, shortening the total length of the lyophilization process to 8–18 h, which significantly reduced energy consumption and production costs. When the mixed lyoprotectant composed of sucrose, trehalose, and mannitol was added to mRNA-LNPs, the eutectic point and collapse temperature of the system were increased. The lyophilized product had a ginger root-shaped rigid structure with large porosity, which tolerated rapid temperature increases and efficiently removed water. In addition, the lyophilized mRNA-LNPs rapidly rehydrated and had good particle size distribution, encapsulation rate, and mRNA integrity. The lyophilized mRNA-LNPs were stable at 2–8 °C, and they did not reduce immunogenicity in vivo or in vitro. Molecular dynamics simulation was used to compare the phospholipid molecular layer with the lyoprotectant in aqueous and anhydrous environments to elucidate the mechanism of lyophilization to improve the stability of mRNA-LNPs. This efficient lyophilization platform significantly improves the accessibility of mRNA-LNPs.

## Introduction

Vaccines are one of the greatest advances in medicine and an important public health tool. Vaccines not only prevent infection, morbidity, and mortality individually but also reduce and eliminate disease prevalence locally, ultimately leading to eradication of disease globally^1^. Of the many COVID-19 vaccines under development^2–4^, the two vaccines that have shown the most promising results in preventing COVID-19 infection represent a new class of vaccine products, namely, vaccines composed of messenger ribonucleic acid (mRNA) strands encapsulated in lipid nanoparticles (LNPs)^5–7^. The efficacy of these mRNA vaccines developed by BioNTech/Pfizer and Moderna is approximately 95%, and they were the first mRNA vaccines to receive emergency use authorization by the Federal Drug Administration (FDA) and conditional approval by the European Medicines Agency (EMA)^8,9^. LNPs composed of lipid-like compounds have shown great promise in delivering mRNA into various cell types in vitro and in vivo to improve uptake efficacy^10–12^. Although mRNA-LNPs show superiority, challenges regarding stability still impede their accessibility^13^. Sub-zero temperature conditions and transportation are needed for the two current licensed mRNA vaccines, namely, Pfizer-BioNTech BNT162b2 (−80 °C to −60 °C) and Moderna mRNA-1273 (−20 °C)^14,15^. The demanding requirements come from the complex interactions among multiple lipid components and the instability of mRNA, which is sensitive to oxygen, moisture, enzymes, and pH^5^. Arteta et al.^16,17^ found that the isotropic LNP core consists of 24% water (volume fraction), and they proposed that mRNA is located inside water cylinders, which are surrounded by cationic lipids. Thus, the mRNA is, at least in part, exposed to water inside the LNPs, which likely contributes to its instability upon storage under non-frozen conditions^18^. Comparable results have been reported by Sebastiani et al.^19^.

As the presence of water initiates degradation reactions in mRNA-LNPs, lyophilization is a logical step to improve the long-term stability of mRNA-LNP formulations^20^. Lyophilization is a process that removes water by sublimation under vacuum at a low temperature^21^. Lyophilization is a relatively mild drying method that improves the stability of vulnerable macrobiomolecules or colloidal nanoparticles^22,23^. However, drying of mRNA-LNPs is more sophisticated as the freezing and the dehydration process induces mechanical force and deforms the vehicle structure, leading to vehicle aggregation, mRNA breakage, or leakage^24,25^. Some studies have shown that although mRNA-LNPs maintain their integrity and encapsulation efficiency, the in vivo uptake efficacy is greatly reduced after lyophilization due to some unknown reasons^15,26–29^. In addition, the reported studies regarding mRNA-LNPs lyophilization mostly focus on the maintenance of its biological activity, while there are few studies on the lyophilization procedures^30^. Lyophilization procedures usually require 40–100 h, and they use a single lyoprotectant^31^, resulting in low production capacity and high costs^32^.

In the present study, we found that the lyophilization procedure and lyoprotectant were related to each other rather than acting independent of each other^33–35^. The suitable lyoprotectant increased the eutectic point and collapsed the temperature of the system. The resulting lyophilized product had a ginger root-shaped rigid structure with large porosity, which tolerated rapid temperature increases and efficiently removed water. Moreover, the suitable lyoprotectant greatly shortened the lyophilization time. In addition, the lyophilized mRNA-LNPs rapidly rehydrated and had acceptable particle size distribution, encapsulation efficiency, and mRNA integrity. The freeze-dried mRNA-LNPs were stable at 2–8 °C, and the freeze-dried samples were redissolved and injected into mice. There was no significant difference in immunogenicity between the freeze-dried samples and the non freeze-dried LNPs. Molecular dynamics simulation was used to compare the phospholipid molecular layer with the lyoprotectant in aqueous and anhydrous environments to elucidate the mechanism of lyophilization to improve the stability of mRNA-LNPs.

## Results

### Lyoprotectant screening

SARS-CoV-2 mRNA was encapsulated in LNPs containing lactose, sucrose, trehalose, mannitol, and glucose protectors, and the mRNA-LNPs were lyophilized by a vacuum freeze-dryer. The sample size after lyophilization and rehydration was used as the evaluation criteria (Table 1). The particle sizes of lactose, mannitol, glucose, and trehalose showed a significant trend compared to before lyophilization, but the particle size of sucrose was increased by approximately 20 nm.

**Table 1:** Screening lyophilization protectants with particle size as the research object.

Number	Protective agent	Size±SD(nm)	PDI ± SD
1	Sucrose	140.2 ± 4.09	0.201 ± 0.35
2	Trehalose	232.9 ± 13.41	0.226 ± 0.23
3	Lactose	367.5 ± 1.363	0.322 ± 0.21
4	Mannitol	529.6 ± 14.12	0.237 ± 0.16
5	Glucose	332.5 ± 2.136	0.339 ± 0.26

### Response surface analysis

According to the response surface, an orthogonal experiment was designed to evaluate the LNP particle size after lyophilization and rehydration based on three factors and three levels of lyophilized protectors (sucrose, trehalose, and mannitol). Multiple regression analysis was conducted using Design-Expert 13.0. The regression equation of size (R) after lyophilization and rehydration with independent variable A (sucrose), independent variable B (trehalose), and independent variable C (mannitol) was as follows:

R = 187.08 + 5.65A + 8.84B + 9.54C + 7.76AB-0.6750AC + 5.5BC-3.67A^2^-9.19B^2^-17.29C^2^

**Fig. 1:**
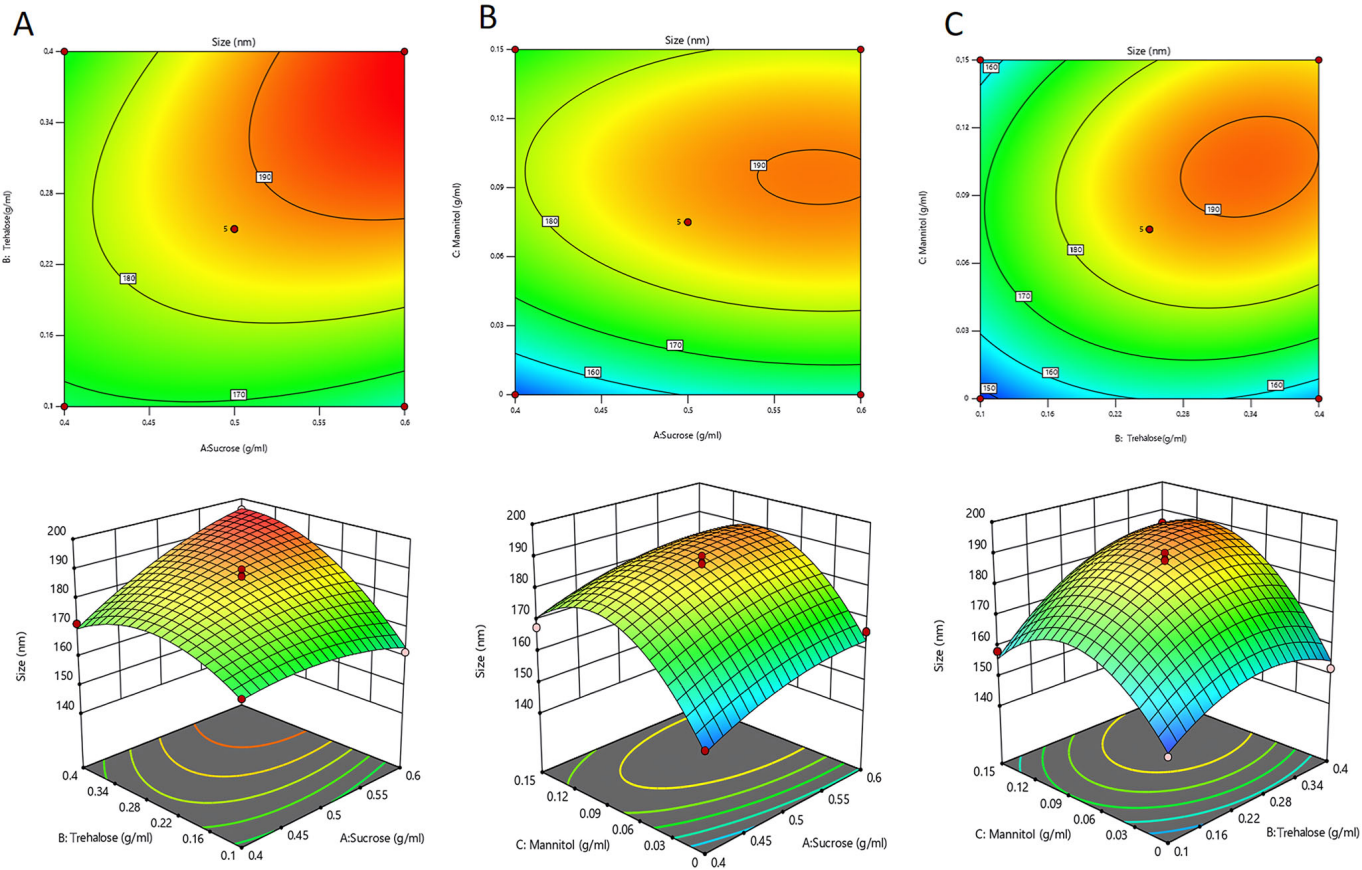
Response surface diagram of the three-factor interaction on particle size after rehydration. **A** Contour plot of interaction between factor A (sucrose) and factor B (trehalose) on particle size. **B** Contour plot of interaction between factor A (sucrose) and factor C (mannitol) on particle size. **C** Contour plot of interaction between factor B (trehalose) and factor C (mannitol) on particle size.

When the relationship between the response value (R) and each factor was described by the regression equation established above, the linear relationship between all independent and dependent variables was significant. The fitting equation R^2^ of the polynomial model was 0.9845, and the coefficient of determination was R^2^_adj_ = 0.9646 after correction, which indicated that the established polynomial model equation had high reliability and that the fitting degree between the predicted value and the actual value was good. ANOVA showed that the model was significant (*P* < 0.001), and the misfit term >0.05 indicated that the misfit term of the model was not significant. Therefore, the regression equation provided a suitable model for selecting the optimal lyophilized protectant. Design-Expert 13.0 was used to generate the response surface contour diagram according to the obtained value (Fig. 1). Independent variables A, B, and C had respective extreme points, which were used to obtain the maximum point of the built surface model. The response surface method was used to determine the optimal formula of the mRNA-LNP composite lyophilized protector containing the appropriate amounts of sucrose, trehalose, and mannitol (Table 2).

**Table 2:** Analysis of variance (ANOVA) of all response variables.

Source	Sum of squares	df	Mean Square	F-value	*p* value	
Model	3767.49	9	418.61	49.38	<0.0001	significant
A-Sucrose	255.38	1	255.38	30.13	0.0009	
B-Trehalose	624.81	1	624.81	73.71	<0.0001	
C- Mannitol	727.71	1	727.71	85.85	<0.0001	
AB	235.62	1	235.62	27.80	0.0012	
AC	1.82	1	1.82	0.2150	0.6569	
BC	121.00	1	121.00	14.27	0.0069	
A²	56.56	1	56.56	6.67	0.0363	
B²	355.60	1	355.60	41.95	0.0003	
C²	1258.71	1	1258.71	148.49	<0.0001	
Residual	59.34	7	8.48			
Lack of fit	39.31	3	13.10	2.62	0.1878	not significant
Pure error	20.03	4	5.01			
Cor total	3826.83	16				

### Physicochemical characterization of lyophilized mRNA-LNPs

**Fig. 2:**
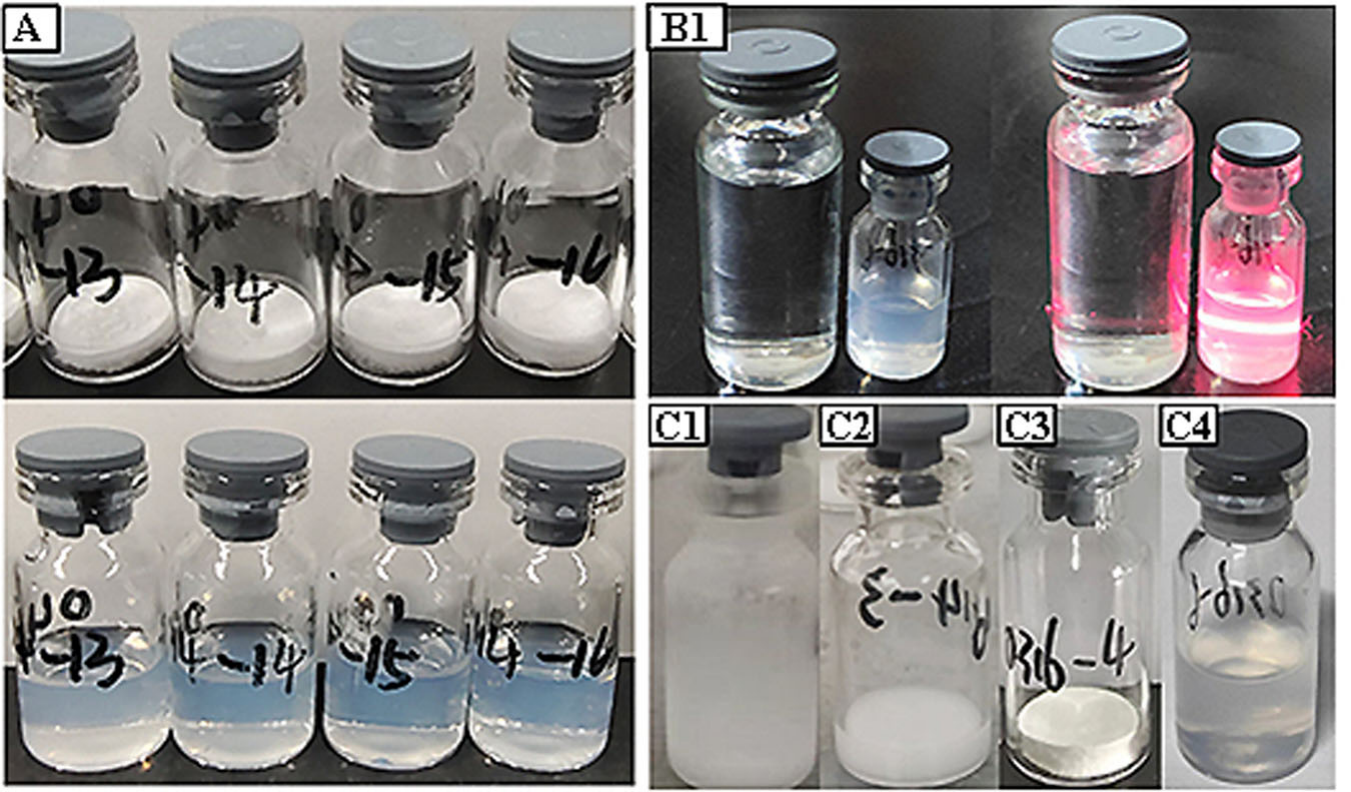
Physicochemical characterization of lyophilized mRNA-LNPs. **A** Lyophilized product (top panel) and rehydrated product (bottom panel). **B** Rehydration morphology of lyophilized product. **C** Samples from the lyophilization LNP process, including (i) pre-freezing, (ii) primary drying, and (iii) secondary drying, as well as (iv) rehydration.

After lyophilized mRNA-LNPs were collected, we examined the appearance of the vials. The lyophilized product in all of the vials had a uniform white smooth surface with no evidence of cake collapse, shrinkage, or structural failure (Fig. 2A). The lyophilized product was quickly dissolved by adding 0.5 mL of nuclease-free water, and the rehydration time of each sample was less than 10 s. After adding water, the vial was gently inverted several times to quickly obtain a clear blue opalescent liquid with a Tyndall effect (Fig. 2B) with a clear light path and no visible solids. The lyophilization method was the direct conversion of liquid to solid through pre-freezing, primary drying, and secondary drying steps (Fig. 2C). Before rehydration, the moisture content of the lyophilized mRNA-LNP was measured, which was approximately 3% (Table 3).

**Table 3:** Determination of moisture content of the lyophilized mRNA-LNP.

Serial number	Water content
1	3.1%
2	2.8%
3	2.9%

### Particle size measurement

**Fig. 3:**
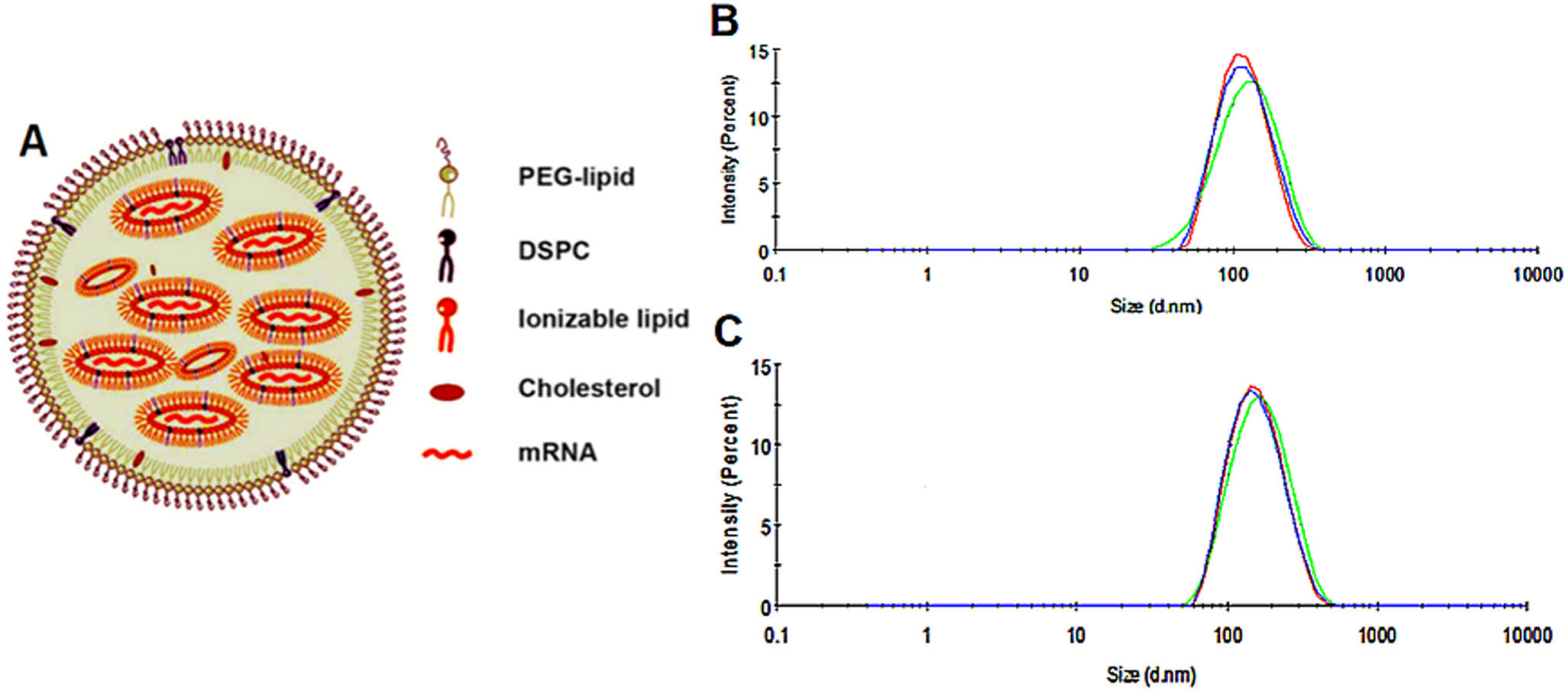
Particle size potentiometry. **A** LNPs were synthesized from PEG-lipid, DSPC, ionizable lipid, and cholesterol at a specific ratio. **B** Particle size of mRNA-LNPs. **C** mRNA-LNP particle size after lyophilization and rehydration.

Figure 3A shows the schematic diagram of mRNA-LNPs. DLS was used to characterize particle size and polydispersity index (PDI; particle size distribution) as the evaluation criteria. The average DLS particle size of the mRNA-LNPs before lyophilization was 113.1 ± 0.1 nm (Fig. 3B), and the PDI was 0.176. Lyophilized mRNA-LNPs stored at 4 °C or lower were quickly dissolved by the addition of nuclease-free water, and measurement of the particle size indicated that the average particle size was increased by 10–20 nm to 121.6 ± 0.2 nm (Fig. 3C). Although the particle size increased, the particle size distribution was narrow with a PDI < 0.2. The freeze-drying procedure did not cause significant damage to the nanostructure of mRNA-LNP, which demonstrated that lyophilization is an excellent method for the long-term storage of mRNA-LNP.

### Microscopic study of lyophilized mRNA-LNPs

**Fig. 4:**
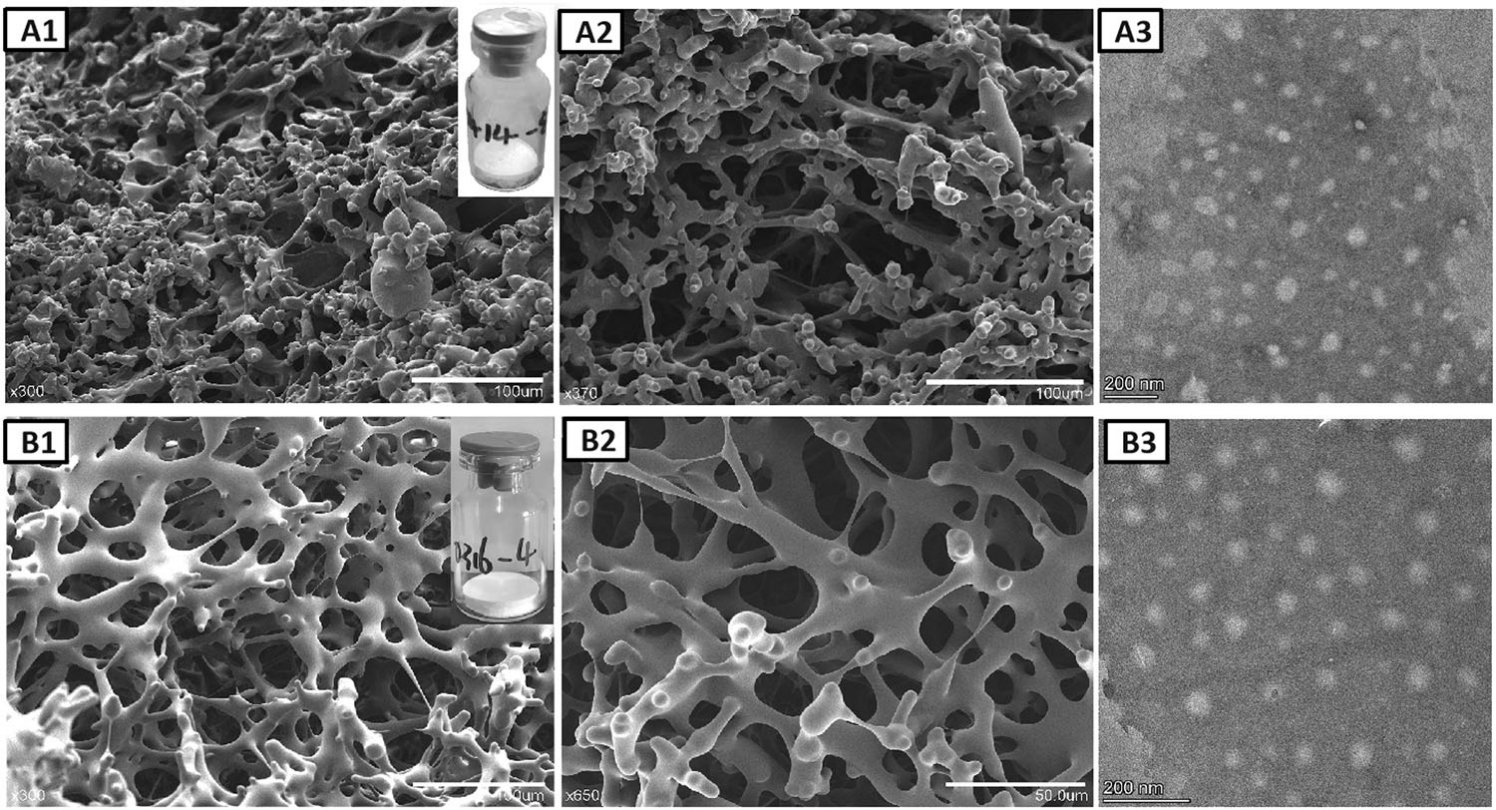
Microstructure characterization of lyophilized product. **A** Scanning electron microscopy (SEM) and transmission electron microscopy (TEM) with the addition of 10% sucrose to lyophilized mRNA-LNPs. **B** Scanning electron microscopy (SEM) and transmission electron microscopy (TEM) images with the addition of sucrose, trehalose, and mannitol to lyophilized mRNA-LNPs. The scale bar length in Figure B2 is 50 μm. The scale bar length in Figure A1, A2 and B1 is 100 μm. The scale bar length in Figure A3 and B3 is 200 nm.

The lyophilized products were visualized with microscopy to examine the microstructure of the lyophilized matrix to demonstrate the conservation of the nanoparticle integrity and to determine whether morphological changes occurred. Many high-resolution microscopy techniques can be used to observe lyophilized nanoparticle formulations, including TEM, cryogenic transmission electron microscopy (cryo TEM), atomic force microscopy (AFM), SEM, and environmental scanning electron microscopy (ESEM)^36^. The microstructure of mRNA-LNPs lyophilized with sucrose, trehalose, and mannitol as well as the microstructure of mRNA-LNPs lyophilized only with sucrose were observed by SEM. The addition of only sucrose to the lyophilized mRNA-LNPs resulted in a lyophilized mRNA-LNP structure containing holes, which indicated that the products collapsed after drying and that the protective structure was not prominent (Fig. 4A). The addition of sucrose, trehalose, and mannitol to the lyophilized mRNA-LNPs resulted in a loose and porous mRNA-LNP structure with a rigid structure, which effectively protected the structure of lyophilized mRNA-LNPs (Fig. 4B).

### Encapsulation efficiency, integrity, and stability of lyophilized mRNA-LNPs

**Fig. 5:**
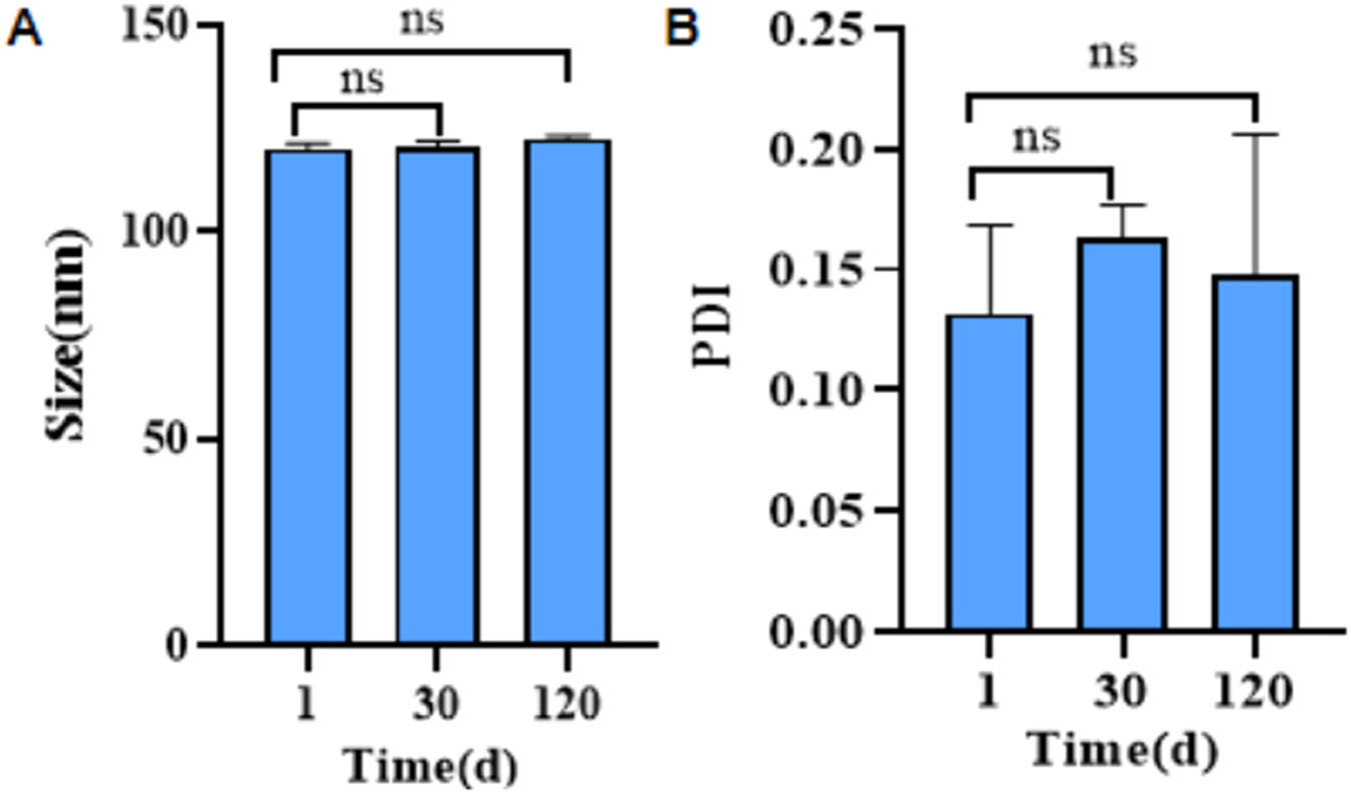
Stability evaluation. **A** Particle size change within 120 days at the storage conditions of 2–8 °C. **B** PDI change within 120 days at the storage conditions of 2–8 °C. Data represented as mean ± SD (*n* = 3). Significance was calculated using the *T* test (ns for *p* > 0.05).

**Fig. 6:**
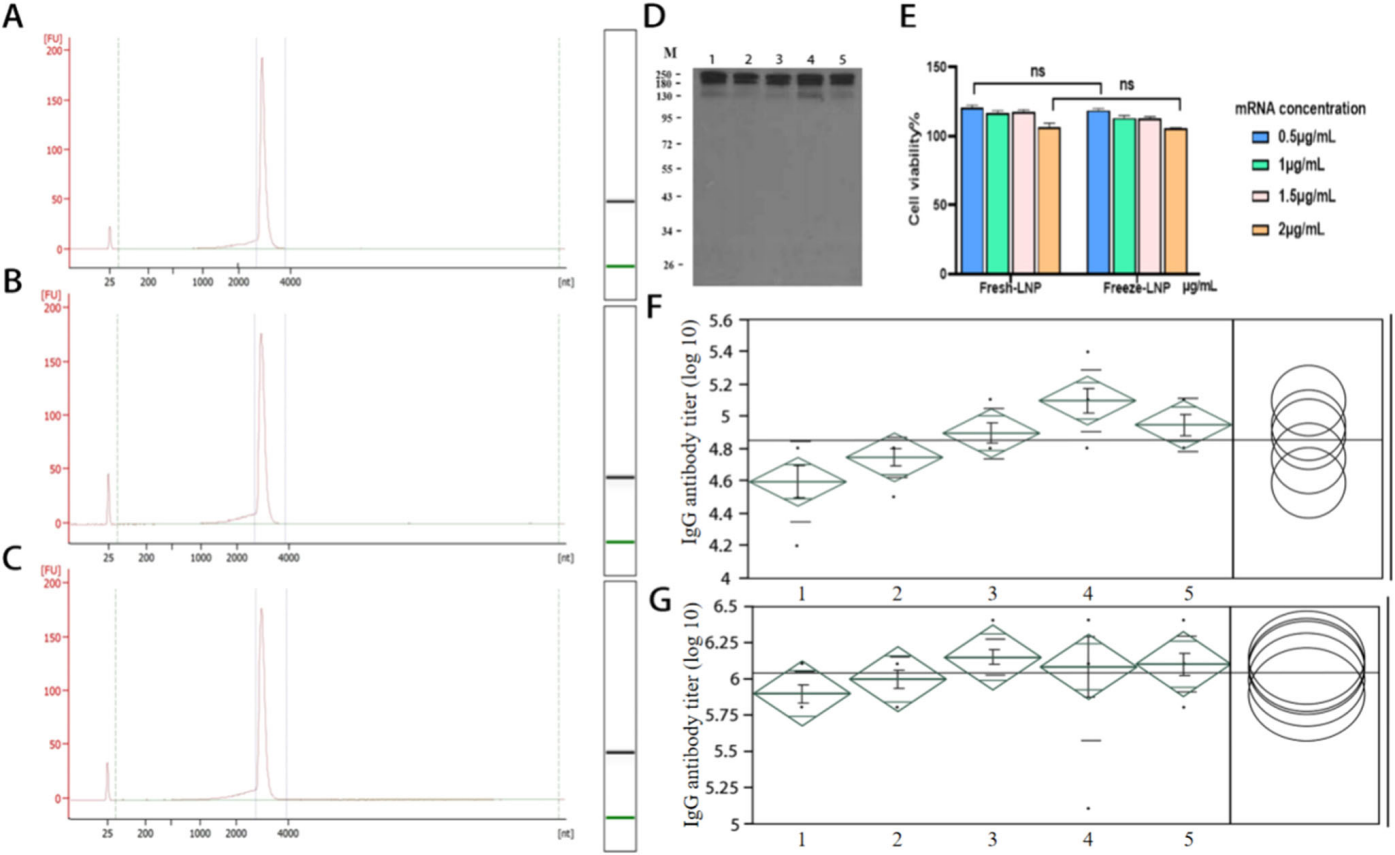
Physicochemical properties of lyophilized mRNA-LNPs. **A** Integrity analysis map of non-lyophilized mRNA-LNPs. **B** Integrity analysis map of lyophilized mRNA-LNP sample 1. **C** Integrity analysis map of lyophilized mRNA-LNP sample 2. **D** Expression of S-protein in mRNA-LNP-treated cells (1,2,3,5-Lyophilized LNP; 4-non-Lyophilized LNP). All blots were processed in parallel and derive from the same experiments. **E** Toxicity evaluation of mRNA-LNPs. **F** Immune effects of lyophilized mRNA-LNPs on Day 14(1,2,3,5-Lyophilized LNP; 4-non-Lyophilized LNP). **G** Immune effects of lyophilized mRNA-LNPs on Day 28(1,2,3,5-Lyophilized LNP; 4-non-Lyophilized LNP). Data represented as mean ± SD (*n* = 3). Significance was calculated using the *T* test (ns for *p* > 0.05).

Lyophilized mRNA-LNPs were placed at 2–8 °C for stability testing for one month, and the particle size and PDI of mRNA-LNPs under storage conditions (2–8 °C) were then determined (Fig. 5A, B). The total amount of mRNA, free mRNA concentration, encapsulation efficiency, and mRNA integrity of lyophilized mRNA-LNPs stored at 2–8 °C were determined using RiboGreen based on the fluorescence signal generated when the RiboGreen dye binds to single-stranded mRNA. The lyophilized samples with the optimal protectant formulation (Samples 1, 2 and 3) showed no significant changes in the encapsulation efficiency of mRNA-LNPs after 4 weeks (encapsulation efficiency of 87.5% at Day 1 and 87.4% at Day 30), which indicated that the lyophilized mRNA-LNPs had good storage stability over 4 weeks. For the non-lyophilized mRNA-LNPs stored at 2–8 °C, the encapsulation efficiency decreased from 93.33 to 86.21%. The integrity of nucleoside-modified mRNA-LNPs was assessed by capillary electrophoresis, which indicated that no significant changes in mRNA integrity were observed for the lyophilized products stored for 4 weeks (Table 4). The integrity of lyophilized mRNA-LNPs was 85% after storage (Fig. 6A, B), but the integrity of the non-lyophilized mRNA-LNPs decreased from 89.4 to 80.2% after storage (Fig. 6C).

**Table 4:** mRNA concentration, encapsulation rate, and integrity.

Sample name	Time(week)	mRNA total concentration (μg/mL)	mRNA concentration of free (μg/mL)	EE (%)	mRNA integrity (%)
mRNA-LNP	0	90.24	6.02	93.33	89.4
	4	84.23	11.62	86.21	80.2
1	0	90.17	6.81	92.45	87.5
	4	90.15	6.84	92.41	87.4
2	0	90.01	8.88	90.13	85.3
	4	89.92	8.92	90.08	84.9
3	0	90.12	7.55	91.62	86.6
	4	90.09	7.68	91.48	86.4

### In vitro evaluation results

The uptake efficiency results of the lyophilized samples indicated that there was no significant difference in the expression of S-protein between lyophilized mRNA-LNPs and non-lyophilized mRNA-LNPs, which indicated that there was no significant difference in the uptake efficiency of cells before and after lyophilization (Fig. 6D). The lyophilization technique did not reduce the cellular biological activity of mRNA‑LNPs. The MTT assay demonstrated that the cell viability of lyophilized mRNA-LNPs and non-lyophilized mRNA-LNPs was more than 110% (Fig. 6E), which demonstrated that the lyophilized mRNA-LNPs had no obvious toxicity.

### In vivo immune effects

Rehydrated lyophilized mRNA-LNPs and non-lyophilized mRNA-LNPs (50 μL) were intramuscularly injected into BALB/c mice for immune efficacy comparison, and the mice were randomly divided into five groups. Serum samples were collected on Days 14 and 28 after primary immunization to detect SARS-CoV-2 specific IgG and neutralizing antibody responses. The immune effects on Day 14 and Day 28 after primary immunization are shown in Fig. 6F, G, respectively. According to the experimental results, both lyophilized and non-lyophilized mRNA-LNPs induced adequate immune responses in vivo. The immune response level of lyophilized preparation No. 3 (Fig. 6G) exceeded that of the non-lyophilized sample on Day 28 after primary immunization (Fig. 6G). These results indicated that the lyophilization process maintains or even improves the immune effect of mRNA-LNPs in vivo.

### Molecular dynamics simulations

Molecular dynamics simulations are used to study the behavior, structure, dynamics, and mechanisms of different types of biomolecules and macromolecules, such as peptides, proteins, lipids, sugars, solvents, and nucleic acids. The results obtained from the molecular dynamics simulation were compared to the experimental data. In vitro and in vivo experiments demonstrated that the encapsulation rate and integrity of lyophilized mRNA-LNPs were not destroyed and that the lyophilized mRNA-LNPs had certain immune ability. We used molecular dynamics simulation to elucidate the mechanism of action between lyophilized protectant and phospholipid membrane. We used DSPC, DSPE and cholesterol as phospholipid molecules to provide self-assembly processes to model phospholipid membrane structures.

**Fig. 7:**
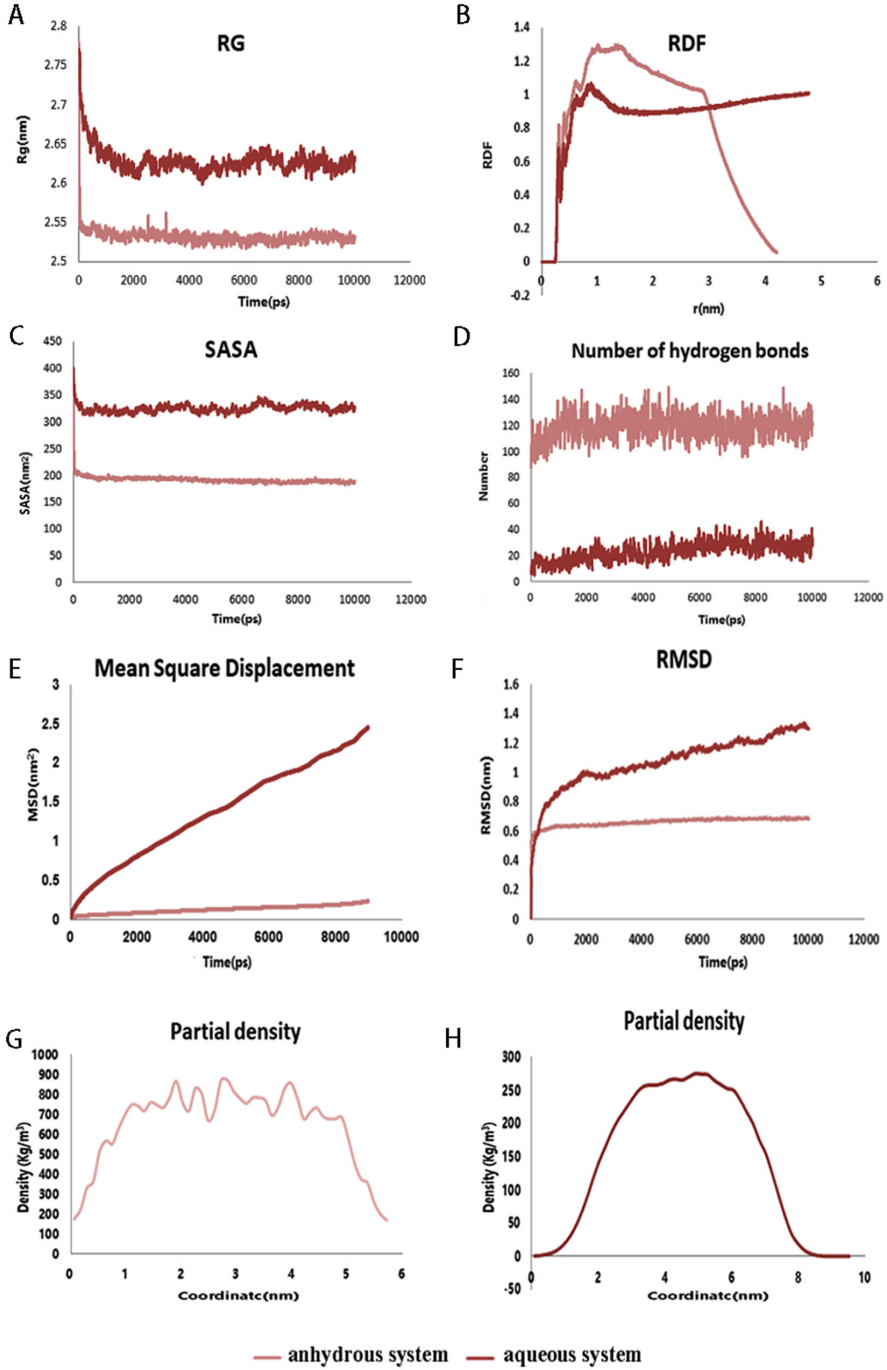
RG, RDF, SASA, Number of hydrogen bonds, MSD, RMSD and partial density in systems. **A** Radius of gyration (RG) of the structure in the simulation system, which was obtained from the simulation trajectory. **B** RDF values of anhydrous and aqueous systems. **C** DSPC/DSPE solvent accessible surface area (SASA) analysis of phospholipid membrane structures formed from cholesterol lipids. **D** Number of hydrogen bonds in the anhydrous system and aqueous system. **E** MSD of the formed phospholipid film. **F** Root mean square deviation (RMSD) of the formed phospholipid film. **G** Density release of the anhydrous system. **H** Density release of the aqueous system.

After running the simulation for 10 ns and calculating a series of indicators, the results of the simulated data were consistent with the experimental results. The RG of a phospholipid molecule is the average distance of all atoms of DSPC, DSPE and cholesterol molecules from the axis of rotation of the phospholipid molecule. In addition, the RG is the standard of molecular compatibility and assembly of phospholipids. Smaller RG values indicate higher system stability. The RG characterizes the compactness of the phospholipid structure. The anhydrous system was demonstrated to be more stable than the aqueous system, and the phospholipid molecules had the lowest RG value at the end of the simulation (Fig. 7A). To study the distribution of sugar molecules in the system, RDF parameters were measured (Fig. 7B). The RDF characterizes the distance relationship between two types of molecules and obtain distance information from sugar molecules to phospholipid molecules. The results showed that in the anhydrous system, the RDF value was the highest at 1–2 nm and tended to be zero with the increase of distance, which indicated that sugar molecules were distributed near phospholipids. However, in the aqueous system, the peak value of RDF was low, and it tended to be equal to 1 with the increase of distance, which indicated that only a few sugar molecules were distributed near phospholipids. Another parameter to explain system formation is SASA (Fig. 7C). SASA is often referred to as the surface of a molecule (e.g. phospholipid molecule) exposed to a solvent (e.g. water) molecule. We evaluated the dynamics of the phospholipid membrane structure by analyzing SASA, which remained at a constant value. The anhydrous system had a lower SASA, which indicated that the phospholipid structure in the anhydrous system was more compact than that in the aqueous system, thus indicating a reasonable simulation. The phospholipid membrane and the lyophilization protective agent formed a certain force, namely, a hydrogen bond. In the aqueous system, there were fewer sugar molecules on the surface of the phospholipid membrane due to the existence of water molecules, which allowed sugar molecules to be accessible in water. Therefore, the number of hydrogen bonds between the sugar and phospholipid membrane was low (approximately 20–30) (Fig. 7D). In the anhydrous system, the phospholipid membrane was coated and protected by the sugar molecules after 10 ns of simulation. The number of hydrogen bonds between the sugar and phospholipid molecules was approximately 140. These findings indicated that it is necessary and reasonable to add lyophilization protectant in the lyophilization process.

The MSD, which is one of the main characteristics of biofilms, increased as the simulation progressed (Fig. 7E). The slope of the curve was greater for aqueous systems compared to anhydrous systems. Analysis of MSD illustrated a lower diffusion rate and higher stability of phospholipid molecules under anhydrous conditions. The RMSD in the simulation process was one of the important indexes in the model trajectory. After least-squares fitting of the selected structures to the reference structures, the positional changes between the conformations during the simulation and the initial conformations were calculated. The slope of the RMSD plot shows the stability of the simulated system, and a slope closer zero indicates a more stable model. If the slope increases gradually or fluctuates sharply, the simulated phospholipid model is more unstable. Comparison of the aqueous and anhydrous systems demonstrated that the slope of the anhydrous system was closer to zero, indicating a more stable system (Fig. 7F). The local density of the box confirms the structure of the phospholipid model. The box density was uniform in the anhydrous system, and the phospholipid model was in a bimolecular membrane structure (Fig. 7G). In the aqueous system, the density was in the middle of the box, and the phospholipid model was globular (Fig. 7H).

**Fig. 8:**
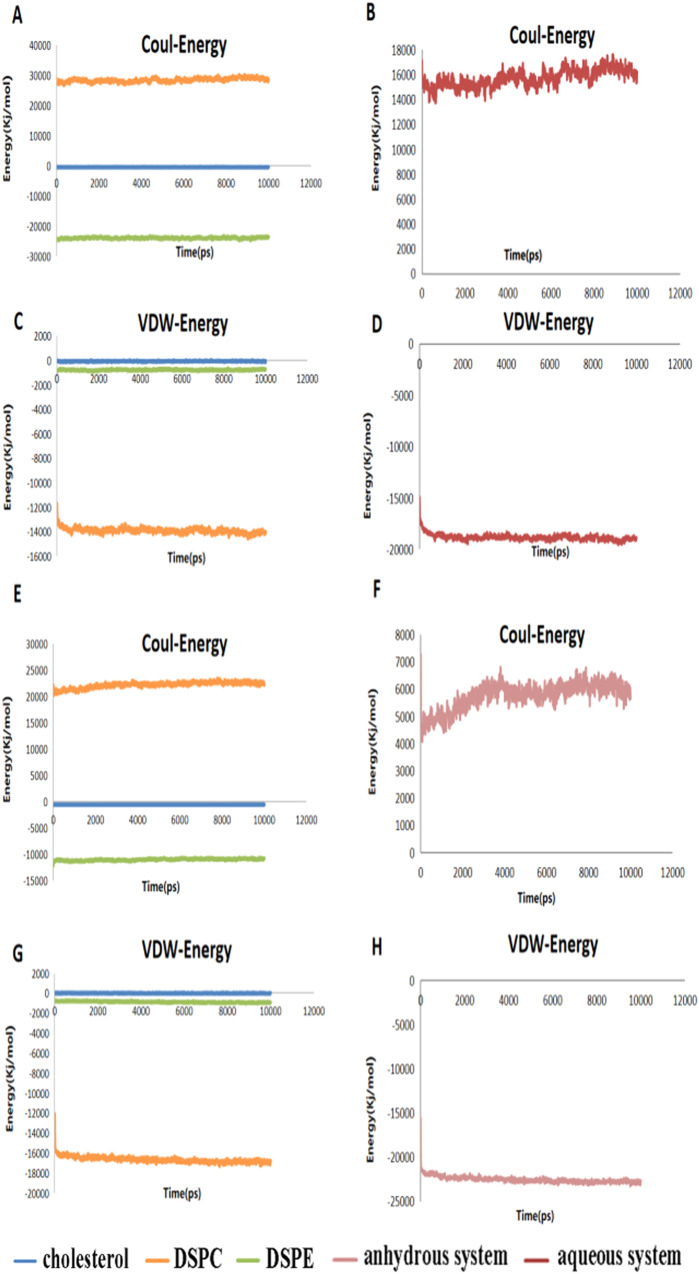
Electrostatic interactions and van der Waals energy in systems. **A** Electrostatic interaction of DSPC, DSPE and cholesterol. **B** Total electrostatic interactions of lipids. **C** Van der Waals interaction of DSPC, DSPE and cholesterol. **D** Total van der Waals interactions of lipids. Electrostatic interactions and van der Waals energy in anhydrous systems. **E** Electrostatic interaction of DSPC, DSPE and cholesterol. **F** Total electrostatic interactions of lipids. **G** Van der Waals interaction of DSPC, DSPE and cholesterol. **H** Total van der Waals interactions of lipids.

The interaction of attractive van der Waals forces and repulsive electrostatics based on the Derjaguin-Landau-Verwey-Overbeek (DLVO) theory directs the stability and formation of colloidal particles (phospholipid membrane). The interaction between van der Waals forces and electrostatics is usually considered a short-range interaction. Van der Waals forces and electrostatic interactions are non-covalent interactions, and although their energy content is low, they significantly influence the formation and stability of phospholipid membrane structures due to their large number. Figure 8 shows that van der Waals interactions played an important role in maintaining structural stability. The van der Waals force in the anhydrous system was more vital than that in the aqueous system. The Coulomb interaction was a repulsive force in the simulation, which increased the system energy, leading to instability.

## Discussion

The emergence of SARS-CoV-2 prompted a global effort to develop protective vaccines. mRNA is an attractive form of the vaccine due to the flexibility in antigen design and development and production^37–41^. However, effective mRNA vaccines require efficient delivery to cells and high antigen expression levels to induce robust immune responses and long-lasting protective immunity^42^. Thus, mRNA vaccines need LNPs to reach their full potential^43^. Although the available data demonstrate the advantages of this promising new platform, the instability of mRNA-LNP vaccines and the need for frozen storage remain major limitations. Stability, which is mainly determined by the mRNA itself and the LNP delivery system, is one of the major challenges in the development of mRNA vaccines. Moderna’s mRNA vaccine (mRNA-1273) needs to be stored and transported at −20 °C, while BioNTech’s mRNA vaccine (BNT162b2) needs to be stored and transported at −70 °C, which undoubtedly increases the difficulty and cost of vaccine storage and transportation. Moderna claims that its mRNA-1237 vaccine can be stored at 2–8 °C for 30 days, while BioNTech’s BNT162b2 vaccine can be stored at 2–8 °C for up to 5 days. However, the detailed stability of the two vaccines at 2–8 °C has not been released. Many traditional vaccines can be stored at 2–8 °C for at least 6 months. However, the two currently approved mRNA vaccines require cold-chain transportation, which greatly limits the transportation and distribution of mRNA vaccines in different countries and regions.

To solve the stability problem of mRNA vaccines, many studies have focused on lyophilization, which significantly improves the stability of mRNA vaccines. However, there is a lack of research on lyophilization procedures and protectants. The lyophilization process generally requires 40–100 h, which is a challenge to mRNA vaccine production capacity and cost. In the present study, we optimized the lyophilization process and identified the optimal lyoprotectant. At the same time, we found that the lyophilization procedure and lyoprotectant were related to each other rather than acting independent of each other. The suitable lyoprotectant increased the eutectic point and collapsed the temperature of the system. The resulting product had a ginger root-shaped rigid structure with large porosity, which tolerated rapid temperature increases and efficiently removed water, allowing the lyophilization time to be shortened. Finally, the highly efficient 8 h lyophilization curve was used for lyophilization (4 h Pre Freezing, 2 h Primary Drying, and 2 h Secondary Drying), and the mixture of sucrose (8.8%), trehalose (2%), and mannitol (0.04%) was used as the optimal lyoprotectant.

In addition, the lyophilized mRNA-LNPs rapidly rehydrated, and the particle size and PDI of the lyophilized LNPs with the optimal lyoprotectant ratio did not change significantly as a result of lyophilization. The particle size of LNPs before lyophilization was approximately 110 nm, and it increased to approximately 121 nm after lyophilization. The PDI was less than 0.2 after lyophilization. Moreover, the morphology of LNPs after lyophilization and rehydration was also uniform and spherical as observed by microscopy. SEM showed that the mRNA-LNPs combined with the three lyoprotectants formed a ginger root-shaped rigid structure with large porosity. The structure tolerated rapid temperature increases and efficiently removed water, which allowed the lyophilization time to be shortened. In addition, the encapsulation rate and integrity of lyophilized mRNA-LNPs were not decreased, and the mRNA-LNPs were stable at 2–8 °C. In vitro and in vivo experiments also showed that the immunogenicity of lyophilized LNPs was not decreased. A molecular dynamics simulation was used to explain the action mechanism of lyophilized protective agent, and good results were obtained.

In the pharmaceutical or food processing industry, the lyophilization process is commonly used to remove moisture, improve product stability, and extend shelf life. In the present study, we investigated the long-term drug stability of these preparations using a long-term stability and high antigenicity efficient lyophilization process. The lyophilized mRNA-LNPs, which do not require for ultra-low temperature storage and transportation, will make mRNA vaccines more convenient to reach every country or region that needs vaccines.

In the present study, we successfully achieved efficient lyophilization of a mRNA-LNP vaccine. The particle size, encapsulation efficiency, PDI, and zeta potential of lyophilized mRNA-LNPs after reconstitution only changed slightly compared to freshly prepared LNPs. In addition, the bioactivity and immunogenicity of the lyophilized mRNA-LNPs were well-maintained, meeting the mRNA vaccine specification. The lyophilized mRNA-LNPs produced high neutralizing antibody titers, and they had long-term storage stability at 2–8 °C, addressing the storage and transportation issue of current mRNA vaccines. The molecular dynamics simulation between the phospholipid membrane and lyophilized protectant in aqueous and anhydrous environments was studied for the first time, and the mechanism of lyophilization improving the stability of the mRNA-LNP system was thoroughly analyzed. In conclusion, lyophilized mRNA-LNPs have high immunogenicity and accessibility, and the lyophilization efficiency is significantly improved by optimizing the lyophilization procedures and protectants.

## Methods

### Materials

The protonable cationic lipid ALC-0315, DSPC, cholesterol and DMG-PEG2000 were purchased from Shanghai Aiwei Top Pharmaceutical Technology Co., Ltd. Ethanol and phosphotungstic acid were obtained from Zhongphenol Chemical Reagent Co., Beijing, China. SARS-CoV-2 S-mRNA was obtained from CanSino Biologics Inc. DMEM, fetal bovine serum, and penicillin streptomycin were purchased from Thermo Fisher Scientific. Glucose, sucrose, trehalose, lactose, mannitol and nuclease-free water were obtained from Tianjin Jiangtian Chemical Co., Ltd. The RiboGreen kits and ELISA kits were from CanSino Biologics Inc.

### Preparation of mRNA-LNPs

LNPs were prepared by microfluidic mixing using the NanoAssemblr Benchtop (Precision Nanosystems, Vancouver, Canada). An ethanolic phase containing lipids was mixed with an acidic aqueous phase (25 mM sodium acetate, pH 4.0) containing mRNA (135 µg/mL) leading to the formation of LNPs^19^. LNPs were produced at a flow rate ratio (aquaous: organic) of 3:1 and a total flow rate of 4.0 mL/min^44^. Ionizable cationic lipid ALC-0315: DSPC: cholesterol: DMG-PEG2000 were used in molar ratio of 49:10:39.5:1.5 to prepare mixed lipid ethanolic phase. Immediately after production, LNPs were dialyzed against an excess of phosphate buffered saline using Slide-a-Lyzer™ dialysis cassettes G2 with a membrane cutoff of 20 kDa for 16–24 h. After dialysis, LNPs were sterilized using 0.22 μm PVDF membrane filters and concentrated to an appropriate volume using Amicon® Ultra-15 centrifugational filter units with a membrane cutoff of 10 kDa at 500–1500 × *g* at 4 °C. Purified LNP was kept at 4 °C and used within 7 days after production^44^.

### mRNA-LNPs lyophilization

SARS-CoV-2 mRNA was encapsulated in mRNA-LNPs containing one or more protective agents, such as lactose, sucrose, trehalose, mannitol and glucose. The mRNA-LNPs were then lyophilized by a vacuum freeze-dryer. The lyophilization cycle was divided into three processes as follows: pre-freezing, primary drying, and secondary drying. At the end of the cycle, the sample was placed under atmospheric pressure, plugged with a stopper, and transferred to a different storage temperature for stability monitoring. All samples were lyophilized, which produced a dense white lyophilized powder^45^.

### Experimental design and statistical modeling

Because the protective agent selected through the pre-test had a poor effect and large particle size, the protective agent needed to be optimized. Thus, we selected sucrose, trehalose, and mannitol as the optimal lyophilized protective agents in the present study. We used the applied mathematical technique of statistical modeling to optimize the lyophilized protective agent considering the influencing factors, and the maximum response with relative significance was obtained. Design Expert 13 software was used to design three levels of three factors as well as to design different doses of protective agents for screening. Statistical Packaging Design Specialist (Version 7.0.0, STAT Ease, Inc., Minneapolis, MN, USA) was used to analyze experimental data and generate regression equations as well as to determine optimal parameters and surface diagrams from response fit plots.

### Rehydration of lyophilized mRNA-LNPs

After adding nuclease-free water to the total mRNA with the target concentration of 0.5 mg/mL, the lyophilized product was quickly rehydrated within 10 s. When nuclease-free water was added, a transparent liquid with blue opalescence was obtained quickly after gently turning the vial several times to entirely and evenly mix the sample.

### Moisture content analysis

In total, 0.1 g lyophilized product was placed into a 831KF Coulometer (Metrohm, Switzerland) to measure the moisture content of the lyophilized sample.

### Morphological study of the lyophilized and rehydrated mRNA-LNPs

The lyophilized and rehydrated mRNA-LNPs were diluted to an appropriate concentration with phosphate buffer, and the sample (10 μL) was placed on a copper net. After 5 min, the samples were stained with 2% phosphotungstic acid and detected by transmission electron microscopy (TEM). Samples for scanning electron microscopy (SEM) imaging were prepared, and an appropriate amount of lyophilized mRNA-LNPs was added onto conductive gel for gold spray treatment and then examined by SEM. Particle size measurements were performed by dynamic light scattering (DLS) after rehydration of the lyophilized product.

### Determination of the stability of lyophilized mRNA-LNPs

The stability of lyophilized mRNA-LNPs stored at different temperatures ranging from 2 to 40 °C for one month was determined^46,47^, and the stability of lyophilized mRNA-LNPs was also determined for storage at 2–8 °C for 4 months. The particle size was measured with a DLS instrument on Days 1, 3, 7, 10, 14, and 30.

### mRNA encapsulation efficiency

We determined and compared the encapsulation efficiency of lyophilized mRNA-LNP ssamples and non-lyophilized samples stored at 2–8 °C for one month^48^. Evaluating mRNA encapsulation efficiency through quantitative RiboGreen assay^49^, which determined the concentration of free and total S-mRNA in LNPs according to the manufacturer’s instructions^50^. The RNA in the LNPs formulation was quantified using the standard curve generated by the corresponding diluted RNA reserve sequence. Both standard and sample were diluted with 1x Tris-EDTA (TE) buffer at pH 8.0, and the target concentration of the final sample was 0.1 ng/μL. Fluorescence was determined using a spectral fluorescence photometer (Varian Cary Eclipse) at an excitation of 500 nm and emission of 525 nm. The standard curve was calculated by linear regression analysis of fluorescence intensity and standard concentration. The RNA encapsulation rate of LNPs samples was determined by comparing the signals of the RiboGreen RNA-bound fluorescent dye in the absence and presence of membrane permeabilization reagent (0.1% Triton X-100). In the absence of a membrane permeabilization reagent, the signal is emitted only from unencapsulated RNA, whereas in the presence of a membrane permeabilization reagent, the signal is emitted from total RNA, including both coated and uncoated RNA^51^. The following formula was used to calculate the encapsulation rate:

$${\rm{Encapsulation}}\; {\rm{efficiency}}\, \% =\frac{{{\rm{Fluorescence}}}_{{\rm{Total}}}\,-\,{{\rm{Fluorescence}}}_{{\rm{unencapsulated}}}}{{{\rm{Fluorescence}}}_{{\rm{Total}}}}\times 100 \%$$

### mRNA integrity

mRNA integrity was measured by capillary electrophoresis using an Agilent 5200 Fragment Analyzer and the Agilent HS RNA Kit (DNF-472-1000). At each time point, LNP samples were treated with Triton X-100 to disrupt the particles, diluted to 0.0025 mg/mL, mixed with the marker diluent, and then heat denatured at 70 °C for 2 min. The unformulated RNA payloads were treated in exactly the same manner. The Fragment Analyzer injected the sample at 7 kV for 150 s with separation at 8 kV for 45 min. Data from each run were analyzed using PROSize 3.0 software. RNA integrity of the formulated mRNA-LNP sample is presented as the percentage relative to the unformulated mRNA standard assayed within the same run.

### Cell culture

Human embryonic kidney cells (HEK293) were donated by Prof. Tao Zhu from Tianjin University of Science and Technology and provided by CanSino Biologics Inc. (Tianjin, China). HEK293 cells were cultured in DMEM containing 10% (v/v) fetal bovine serum (FBS) in a humid atmosphere of 37 °C and 5% CO_2._

### In vitro cytotoxicity of lyophilized mRNA-LNPs

The cell viability after treatment with lyophilized mRNA-LNPs and non-lyophilized mRNA-LNPs was evaluated by a MTT assay. In brief, 5×10^4^ HEK-293 cells per well were seeded into a transparent 96-well plate. After 24 h of culture, lyophilized samples and non-lyophilized samples (0.5, 1, 1.5 and 2 μg/mL mRNA concentration) were added followed by incubation at 37 °C and 5% CO_2_ for 24 h. Then, 10 μL of nuclease-free water and 90 μL of complete medium were mixed and added to the wells. After 24 h, 20 μL of MTT was added to each well followed by incubation at 37 °C and 5% CO_2_ for 3 h. The cell culture medium was removed, and 200 μL of DMSO was added to dissolve the crystals with shaking for 20 min. A microplate reader was used to measure the absorbance at 570 nm, and the background value measured at 680 nm was used for calibration. The average percentage of living cells compared to untreated cells represented 100% viability.

### Western blot analysis

The expression of mRNA-LNPs was analyzed by western blot analysis. Whole-cell lysates and supernatants were combined with a loading buffer containing dithiothreitol, separated by 10% SDS-PAGE, and transferred onto a polyvinylidene difluoride (PVDF) membrane using a semidry blotting apparatus (15 V and 60 min). The membrane was blocked with nonfat milk in a PBS buffer containing 0.5% Tween-20. Samples were incubated with a primary antibody (SARS-CoV-2 S1 polyclonal antibody at 0.1 µg/mL, CanSino Biologics, China) for 1.5 h followed by incubation with a secondary goat anti-rabbit IgG-HRP for 45 min. The membrane was incubated with a western blot substrate (Solarbio, China), and images were acquired using an ECL system (CLINX, China).

### Animals

Female BALB/c mice aged 6–8 weeks and weighing 18–22 g were used in the experiment. The protocols and procedures used in animal experiments were reviewed and approved by the Ethics Committee of CanSino Biologics Inc. (Tianjin, China).

### In vivo immune effect

Rehydrated lyophilized mRNA-LNPs and non-lyophilized mRNA-LNPs (50 μL) were intramuscularly injected into BALB/c mice for immune efficacy comparison, and the mice were randomly divided into five groups (four groups for lyophilized mRNA-LNPs of different freeze drying procedure, one group for non-lyophilized mRNA-LNPs). Serum samples were collected on Days 14 and 28 after primary immunization to detect SARS-CoV-2 specific IgG and neutralizing antibody responses.

Epitope-specific antibody titers were detected by a semi-quantitative enzyme-linked immunosorbent assay (ELISA). Briefly, recombinant SARS-CoV-2 RBD protein (2.0 μg/mL) was coated on a 96-well high-binding polystyrene assay plate. After incubating overnight at 4 °C, plates were blocked with 2% bovine serum albumin (BSA) for 4 h at room temperature and washed three times with wash buffer. Subsequently, serial dilutions of the sera samples were added and incubated overnight at 4 °C. After washing five times with wash buffer, goat anti-mouse IgG-HRP (1:50000 dilution) was added to the plate and incubated for 2 h at 37 °C. The trimethyl borane (TMB) solution (Solarbio) was added, and the plates were incubated for 30 min in the dark. Finally, 2 M H_2_SO_4_ was applied to stop the reactions, and the absorbance at 450 nm was acquired using an ELISA reader.

### Molecular dynamics simulations

**Fig. 9:**
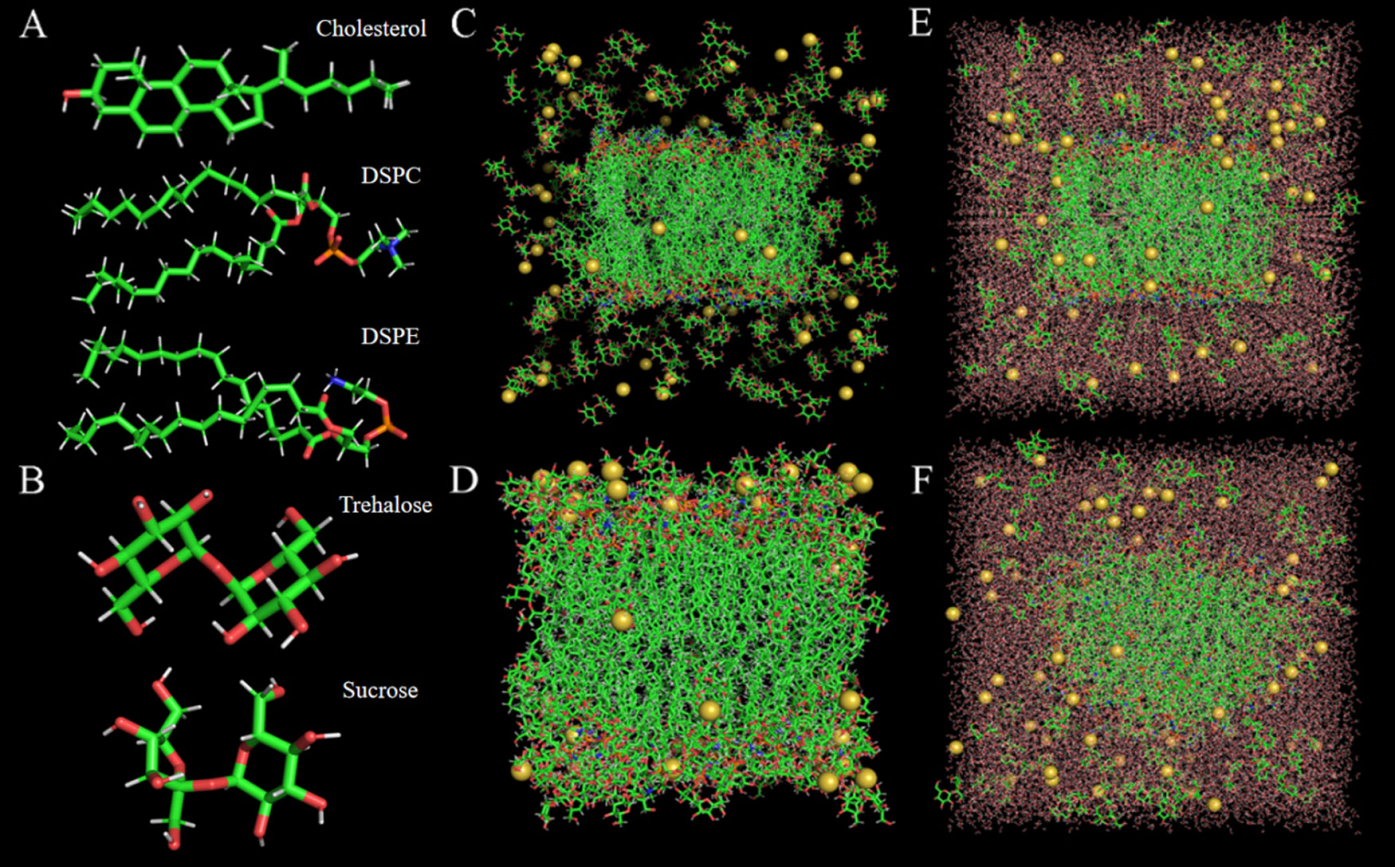
All-atom models. **A** All-atom models of the phospholipid bilayer composed of three phospholipids, namely, DSPC, DSPE and cholesterol. **B** All-atom models of sucrose and trehalose with the polyglycan modeler. **C** Molecular dynamics morphology after equilibrium in anhydrous system. **D** The molecular dynamics morphology was simulated for 10 ns in an anhydrous system. **E** Molecular dynamics morphology after equilibrium in an aqueous system. **F** The molecular dynamics morphology of an aqueous system was simulated for 10 ns (yellow beads indicate NaCl molecules used in equilibrium).

According to the formulation, DSPC, DSPE, sucrose and cholesterol were selected for molecular dynamics simulations (Fig. 9A, B). The software used for the molecular dynamics simulations was GROMACS 2020.6^52^, and Charmm-Gui was used to build two all-atomic models, in which phospholipid molecules, sucrose molecules, trehalose molecules, phospholipid type, DSPC amount, DSPE amount and cholesterol amount were added in proportion^53^. Due to the need to simulate the state of LNPs after lyophilization, we constructed the following two systems: an aqueous system and an anhydrous system (Fig. 9C–F). The aqueous system included a TIP3P water molecule model. Both atomic models were placed in a box (size of 10 nm × 10 nm × 10 nm) with periodic boundary conditions. In addition to phospholipids and lyoprotectants, the remaining water molecules filled the box in the aqueous system. The cut-off radius of the Lennard Jones and van der Waals interaction was 1.4 nm. The step method reduced the energy of all atomic models to 1000 steps. Finally, the model was simulated with 10 ns molecular dynamics. All atomic models were equilibrated in situ at 0.5 ns and 1 atm pressure of 303.15 K (NPT and NVT). For both systems, the simulations were performed at constant temperature and volume (NVT), and periodic boundary conditions were applied to stabilize the simulated space. The force field used was CHARMM 36. The resulting analysis included the electrostatic effect, van der Waals effect, total energy, number of hydrogen bonds, density, radial distribution function (RDF), solvent accessible surface area (SASA), root mean square deviation (RMSD), radius of gyration (RG), and mean square displacement (MSD)^46^. These analyses helped investigate the protection mechanism of the sugar agents during the lyophilization of phospholipids, and an energetic analysis of the system was performed to determine the stability of the phospholipid structure.

### Reporting summary

Further information on research design is available in the Nature Research Reporting Summary linked to this article.

### Supplementary information

Supplementary Information

Reporting Summary

## Data Availability

The datasets used and/or analyzed during the current study are available from the corresponding author on reasonable request.
