# Supplementary material for: Lyophilization process optimization and molecular dynamics simulation of mRNA-LNPs for SARS-CoV-2 vaccine
Source: NPJ Vaccines. 2023 Oct 9;8:153. doi: 10.1038/s41541-023-00732-9 (PMC10562438; doi:10.1038/s41541-023-00732-9)
Supplement: Supplementary file 1 — Supplementary Information [file 41541_2023_732_MOESM1_ESM.pdf]

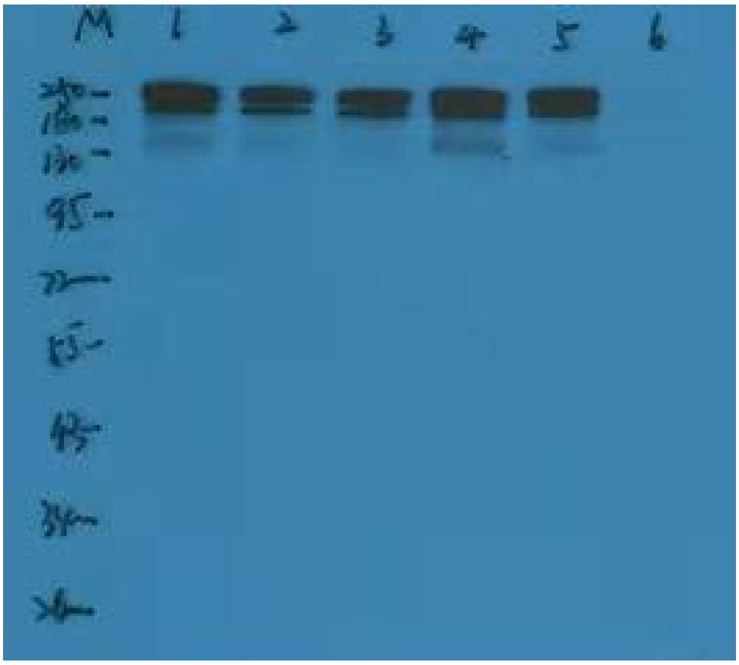

Figure S1 The the original western blots image of Figure 6D. Expression of S-protein in mRNA-LNP-treated cells (1,2,3,5-Lyophilized LNP;4-non-Lyophilized LNP;6-negative control). All blots were processed in parallel and derive from the same experiments.
